# Supplementary material for: Epsin and Sla2 form assemblies through phospholipid interfaces
Source: Nat Commun. 2018 Jan 23;9:328. doi: 10.1038/s41467-017-02443-x (PMC5780493; doi:10.1038/s41467-017-02443-x)
Supplement: Supplementary file 1 — Supplementary Information [file 41467_2017_2443_MOESM1_ESM.pdf]

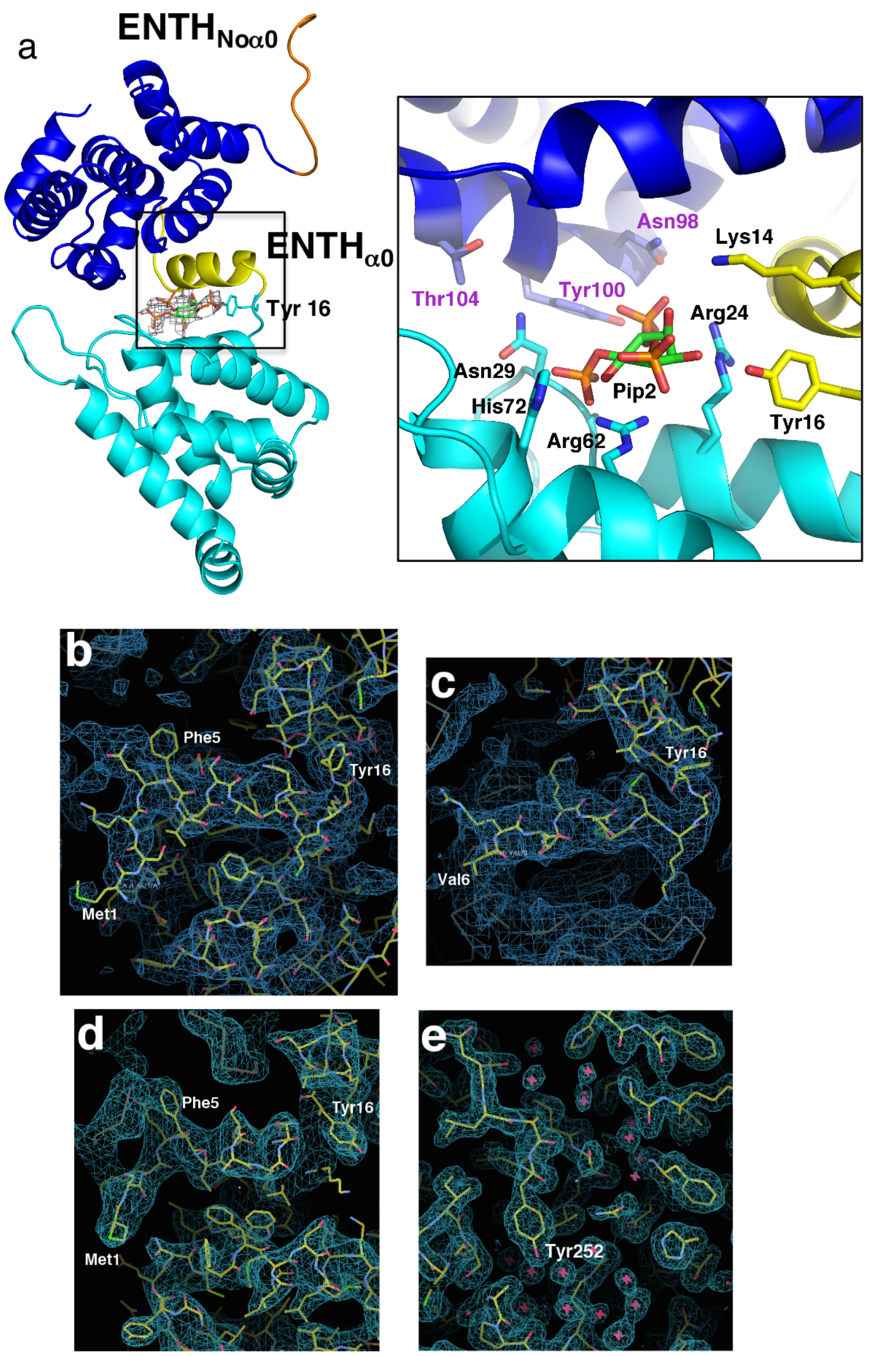

Supplementary Figure 1 │PIP2 binding scheme and binding mode between two ENTH2 molecules. a, Left panel: An electron density omit map at 2.5 σ level is displayed around the phosphoinositol group of the PIP2 molecule situated between two protomers of the ENTH2 domain. One protomer (cyan) contains a folded α0 helix

(yellow). The other protomer (dark blue) contains an unfolded N- terminus pointing away from the PIP2 binding site. Right panel: Zoom of the PIP2 binding site. Ribbon diagram of the surrounding of the PIP2 molecule (in sticks), with $ENTH_{\alpha0}$ in cyan (except for the α0 helix, which is in yellow), and the $ENTH2_{No\alpha0}$ in dark blue. Residues that have side chains within 5 Å of PIP2 are shown as sticks. b) 2mFo-DFc map at 2σ contour level of the α0 helix in the ENTH2/PIP2 crystal structure. c) 2mFo-DFc map at 2σ contour level of the N-terminal region of the $ENTH2_{No\alpha0}$ molecule in the ENTH2/PIP2 crystal structure. d) 2mFo-DFc map at 2 σ contour level of the α0 helix in the ENTH1 crystal structure. e) 2mFo-DFc map at 1 σ contour level of the region around Tyr252, the conserved insertion observed in the Sla2 ANTH crystal structure.

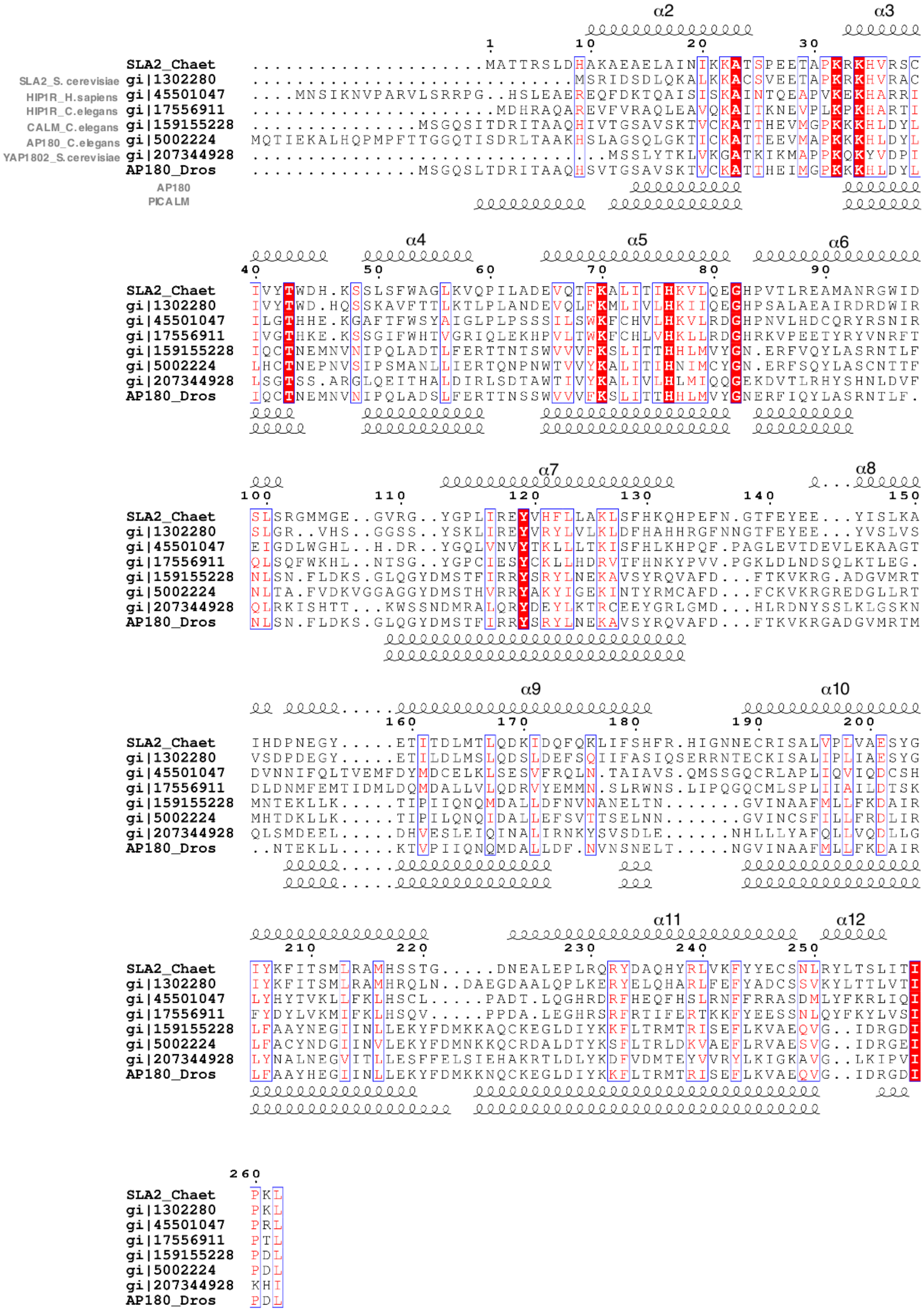

Supplementary Figure 2 │Structure-based sequence alignment of the ANTH domains of members of the Hip1R/Sla2 subfamily (top) and CALM subfamily (bottom). The secondary structure of Hip1R/Sla2 ANTH (from *C. thermophilum*) is shown on top of the alignment with helices depicted as curls and annotated according

to Ford et al[1], using DSSP[2] to assign secondary structure. For comparison, the secondary structures of AP180 and human CALM are shown below the alignment.

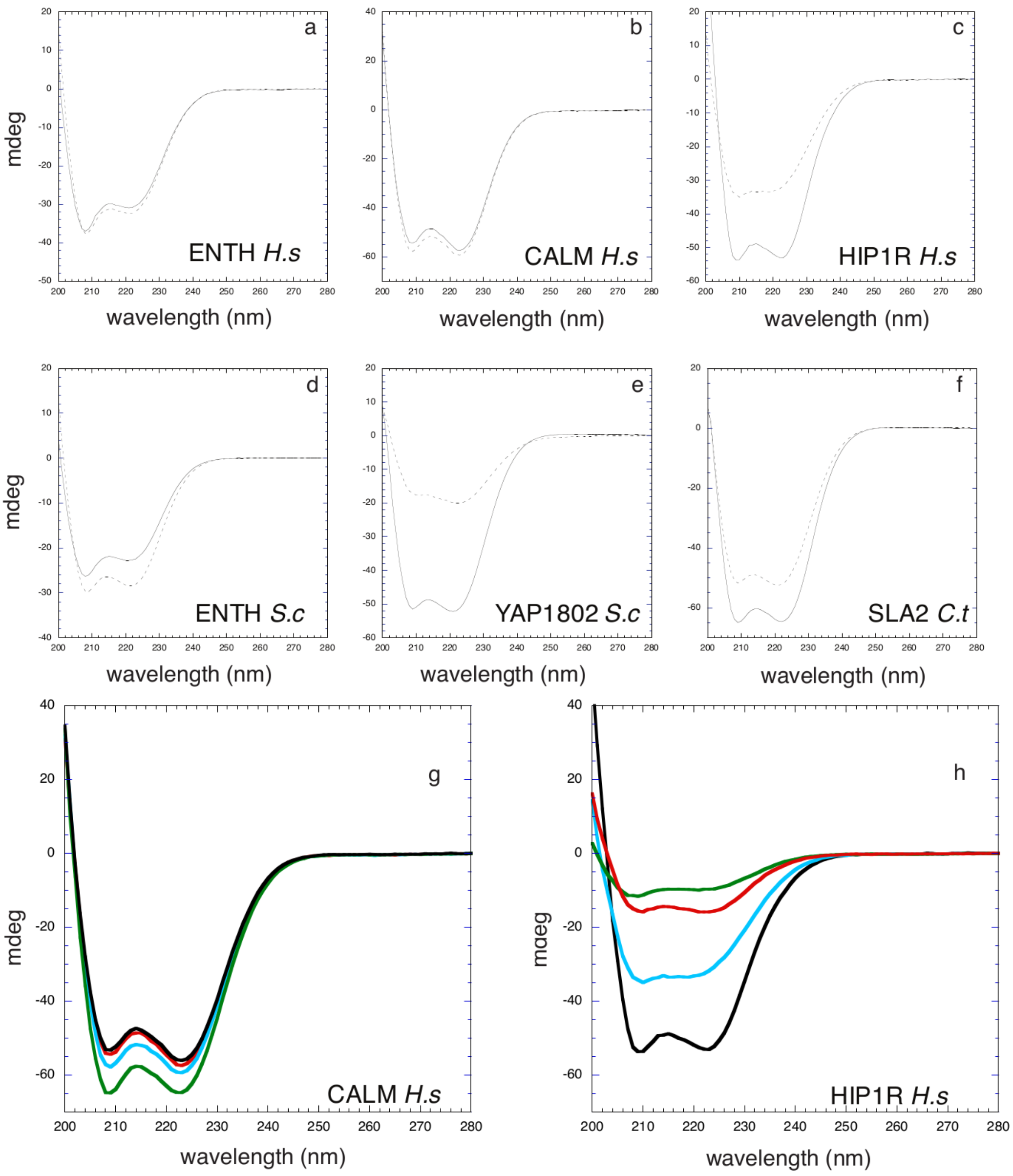

Supplementary Figure 3 │Far UV-CD spectra of ANTH and ENTH domains in the absence (full line) or presence of 200 µM PIP2 (dotted line). a, human epsin-1 ENTH (ENTH *H.s*); **b,** human CALM ANTH (CALM *H.s*); **c,** human Hip1R ANTH (Hip1R *H.s*); **d,** *S. cerevisiae* Ent2 ENTH (ENTH *S.c*); **e,** *S. cerevisiae* Yap1802 ANTH (YAP1802 *S.c*); **f,** *C. thermophilum* Sla2/Hip1R ANTH (SLA2 *C.t*). The loss in CD raw signal is a combined effect of the loss of secondary structure and protein

aggregation caused by addition of the phospholipid. **g**, Human CALM and **h**, human Hip1R ANTH domains in an amphipathic environment. Proteins measured in 10mM TrisHCl pH 8.0 and 250 mM NaCl – without- (black line), and with 200 μM PIP2 (red line), with 0.1mM DDM and 50 μM PIP2 (pale blue line), or with 0.5 mM DDM (3 higher than its CMC) (green line).

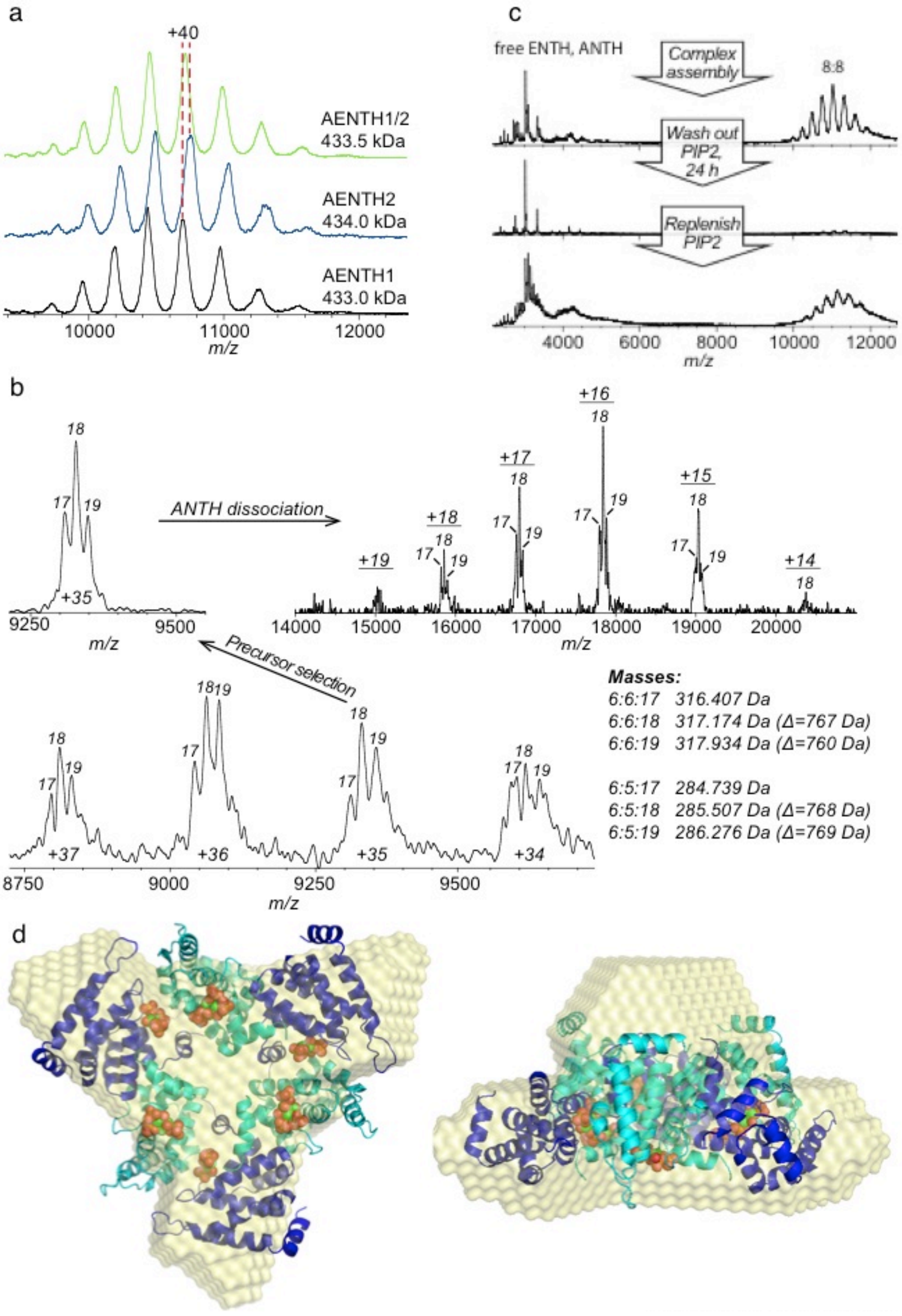

Supplementary Figure 4 │Dissecting the assembly pathways of ENTH/ANTH$_{Sla2}$/PIP2 complexes. a, Native MS shows that ENTH/ANTH$_{Sla2}$/PIP2 8:8 complexes from *S. cerevisiae* can be assembled with mixed ENTH1 and ENTH2 core *in vitro*. Due to different molecular weights (Δm=111 Da) of the used constructs

of ENTH1 and ENTH2, 8:8 ENTH/ANTH$_{Sla2}$/PIP2 complexes with the different ENTH forms have different masses. The ENTH1/ANTH$_{Sla2}$/PIP2 complex (black) has a 1 kDa higher mass than the ENTH2/ANTH$_{Sla2}$/PIP2 complex (blue). When ENTH domains were mixed in an equimolar ratio and complexes with PIP2 and Sla2 ANTH were assembled an intermediate mass (green) is observed. There seems to be no preference for either ENTH variant suggesting similar binding affinities for complex formation. The peak tops of the +40 peaks from the pure ENTH1 or ENTH2/ANTH$_{Sla2}$/PIP2 are indicated highlighting the intermediate position of the mixed form. **b,** Dissociation of the *S. cerevisiae* 6:6 ENTH2/ANTH$_{Sla2}$ protein complex. Native mass spectra from *S. cerevisiae* 6:6 ENTH2/ANTH$_{Sla2}$ protein-phospholipid complexes show a fine structure revealing varying numbers of PIP2 molecules in the complexes. Using the measured masses of *S. cerevisiae* domains ENTH2 and ANTH$_{Sla2}$ and the theoretical mass of PIP2, the precise stoichiometry was calculated and showed 17 to 19 PIP2 molecules. The mass difference (Δ) to the species with one less PIP2 molecule was calculated and listed. The +35 charge state was selected as a precursor for MS/MS experiments. Increased collision voltages led to dissociation of one ANTH$_{Sla2}$ domain, masses of the remaining 6:5 ENTH2/ANTH$_{Sla2}$ complexes were measured showing unaltered lipid stoichiometry and ratio. Still, 17 to 19 PIP2 molecules were found in the complexes, suggesting a protected localization of PIP2 molecules in the core. **c,** Reversible *S. cerevisiae* ENTH/ANTH$_{Sla2}$/PIP2 complex formation. Complexes were assembled and a native mass spectrum was recorded before PIP2 was dialyzed out of the buffer. After 24 h the samples contained mainly monomeric ENTH and ANTH proteins and only little complexes. Upon addition of PIP2, ENTH/ANTH$_{Sla2}$/PIP2 complexes were reformed. **d,** Superposition of SAXS refined rigid body models (cartoon) and the reconstructed

ab initio envelope (yellow semi-transparent surface) of the ENTH hexamer is shown. P3 symmetry was enforced and the tandem ENTH domains with bound PIP2 used as rigid bodies are shown in blue and cyan same as in Extended Data Figure 1. The right panel shows a 90° rotation of the model.

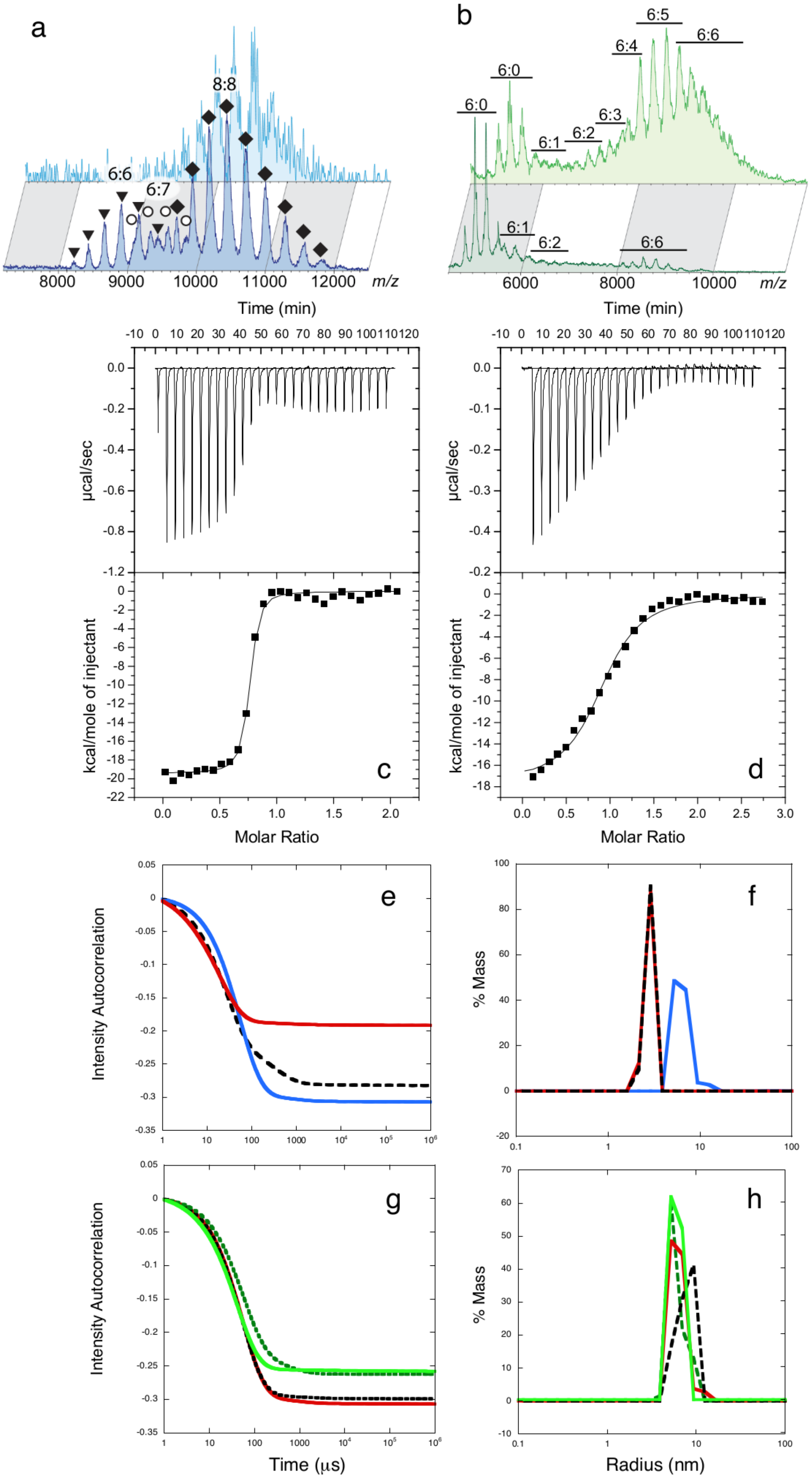

a
8:8
6:6
6:7
8000
9000
10000
11000
12000
m/z
b
6:0
6:0
6:1
6:2
6:3
6:4
6:5
6:6
6:1
6:2
6:6
6000
8000
10000
m/z
Time (min)
µcal/sec
kcal/mole of injectant
Molar Ratio
c
d
Intensity Autocorrelation
e
f
% Mass
g
h
Time (µs)
Radius (nm)

Supplementary Figure 5 │PIP2 driven cross-species interaction of ENTH and $ANTH_{Sla2}$ domains. **a,** Cross-species complex assembly of ENTH/ANTH/PIP2 complexes analyzed by native MS. Mixing of ENTH (*C. thermophilum*) and Sla2 ANTH (*S. cerevisiae*) shows 6:6, 6:7 and 8:8 complexes (dark blue). Formation of ENTH2 (*S. cerevisiae*) and Sla2/Hip1R ANTH (*C. thermophilum)* yielded poor mass spectra, the *m/z* range of the peak series suggests formation of 8:8 complexes (light blue). **b,** Sla2/Hip1R ANTH proteins from *S. cerevisiae* (dark green) and *C. thermophilum* (light green) were added to human epsin-1 ENTH and PIP2. In both cases ANTH proteins bind to the human ENTH 6-mer in different stoichiometries. No complexes larger than 6:6 are observed. **c,** Isothermal titration calorimetry (ITC) on the interaction of ANTH and ENTH2 from *S. cerevisiae* in the presence of 200 μM PIP2. **d,** ITC of ANTH from *C. thermophilum* and ENTH2 *S. cerevisiae* in the presence of 200 μM PIP2. **e** and **f**, Dynamic light scattering (DLS) of the ANTH and ENTH2 (*S. cerevisiae*) interaction in the absence of PIP2 (red); with 200 μM PIP2 (blue) and for the Sla2 ANTH mut (Extended data, constructs) with 200 μM PIP2 (doted line black). **e**, Autocorrelation functions. **f**, Percentage of mass as a function of the hydrodynamic radius (nm). Only the ENTH/ANTH interaction in the presence of PIP2 shows a peak from a macromolecular complex (blue). Note that the red and black lines overlap. **g** and **h**, DLS of the ANTH and ENTH interactions in the presence of 200 μM PIP2: ENTH2/ANTH from *S. cerevisiae* (red); ENTH1/ANTH from *S. cerevisiae* (dotted line black); ANTH from *C. thermophilum* and ENTH2 from *S. cerevisiae* (dotted dark green) and ANTH from *C. thermophilum* and ENTH1 *S. cerevisiae* (light green). **g,** Autocorrelation functions. **h,** Percentage of mass as a function of the hydrodynamic radius (nm). All cases show peaks corresponding to the macromolecular complexes.

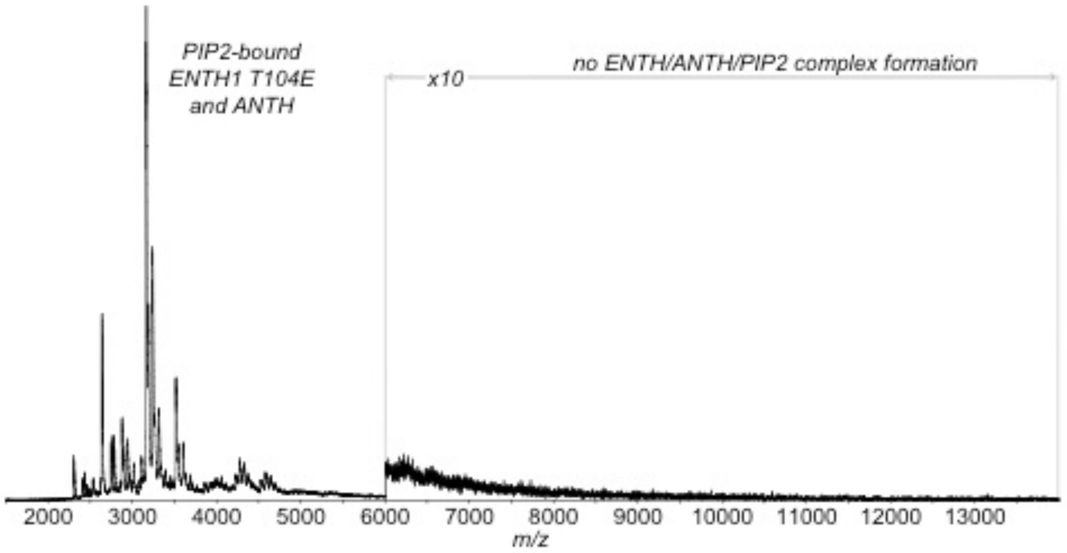

Supplementary Figure 6 │ *S. cerevisiae* epsin Ent1 ENTH T104E domains do not form ENTH/ANTH/PIP2 complexes. *S. cerevisiae* epsin Ent1 ENTH T104E and Sla2 ANTH were mixed with PIP2, but no complex formation was observed in native MS. The mass range above 6000 *m/z* is 10x magnified to highlight the absence of large complexes. PIP2 bound and unbound ENTH T104 and ANTH domains can be found in the lower mass range.

Supplementary Table 1: K_D values (standard deviation, N=3) of PIP2 binding to different ENTH and ANTH domains determined by native MS. Unless stated differently, measurements were performed in 300 mM NH_4OAc pH 8.0. Samples marked with an asterisk were measured at the more physiological concentration of 160 mM NH_4OAc pH8.0.

**Protein**	**Organism**	**K_{D1} /µM**	**K_{D2} /µM**
Ent1 ENTH	*S. cerevisiae*	83 (20)	65 (2)
Ent1 ENTH*	*S. cerevisiae*	100 (50)	52 (6)
Ent1 ENTH T104E	*S. cerevisiae*	280 (80)	450(110)
Ent2 ENTH	*S. cerevisiae*	100 (10)	82 (21)
ENTH	*C. thermophilum*	74 (23)	200 (40)
Sla2 ANTH	*S. cerevisiae*	158 (19)	130 (90)
Sla2 ANTH*	*S. cerevisiae*	110 (2)	160 (50)
Sla2 ANTH	*C. thermophilum*	211 (7)	270 (80)
Sla2 ANTH mut	*S. cerevisiae*	300 (70)	-
CALM ANTH	*H. sapiens*	44 (8)	125 (14)
CALM ANTH*	*H. sapiens*	45 (8)	157 (20)

Supplementary Table 2: Molecular weights (*M*) of single ENTH and ANTH proteins or ENTH/ANTH complexes from *S. cerevisiae*, *C. thermophilum* and *H. sapiens* were determined from at least three MS or MS/MS measurements and listed with standard deviations and the average full width half maximum (FWHM), all values in Da. Using the experimental masses of single ENTH and ANTH proteins, an expected protein mass for the complexes was determined. By subtracting the expected protein mass from the measured mass, the lipid mass of the complex was calculated. Division of the lipid mass by the theoretical PIP2 mass (741.25 Da) lead to an estimated number of PIP2 molecules. Variations of the PIP2 content were calculated based on the FWHM value. Results of 6:6 and 8:8 complexes from MS measurements show broader peaks due to remaining buffer molecules, resulting in a larger FWHM value and a slight overestimation of the number of PIP2 molecules. For *H. sapiens* 6:0 and 5:0 ENTH complexes resolution was sufficient to directly determine the exact number of phospholipids.

**Protein**	**Species**	M_{Theo}	M_{Exp}	**St.dev.**	**FWHM**
ENTH1	*S. cerevisiae*	18847.4	18846.8	0.6	8.8
ENTH2	*S. cerevisiae*	18958.4	18957.6	3.5	10.0
Sla2	*S. cerevisiae*	31638.1	31626.7	9.8	16.7
Sla2 *	*S. cerevisiae*	31638.1	31635.8	0.6	23.3
ENTH	*C. thermophilum*	18041.6	18168.0	5.4	10.9
Sla2	*C. thermophilum*	30407.8	30263.3	0.6	14.8
Sla2 *	*C. thermophilum*	30407.8	30263.4	4.0	27.0
ENTH	*H. sapiens*	18311.8	18571.4	1.0	11.2
ENTH *	*H. sapiens*	18311.8	18570.3	0.4	13.2
Hip1R	*H. sapiens*	34996.3	34997.5	1.9	16.0
Hip1R *	*H. sapiens*	34996.3	34994.4	0.5	23.7
CALM	*H. sapiens*	32894.8	32990.8	16.4	181.5
**Complex**	**Species**	***N* PIP2**	M_{Exp}	**St.dev.**	**FWHM**
6:6 ENTH1/ANTH	*S. cerevisiae*	19±2	317332	209	1660
8:8 ENTH1/ANTH	*S. cerevisiae*	25±3	422574	298	2373
8:7 ENTH1/ANTH *	*S. cerevisiae*	23±1	389639	134	701
6:6 ENTH2/ANTH	*S. cerevisiae*	19±2	317727	356	1119
8:8 ENTH2/ANTH	*S. cerevisiae*	24±3	422790	240	2385
8:7 ENTH2/ANTH *	*S. cerevisiae*	23±1	389897	87	935
6:6 ENTH/ANTH	*C. thermophilum*	18±3	304232	345	2406
8:8 ENTH/ANTH	*C. thermophilum*	25±4	405983	247	2720
8:7 ENTH/ANTH *	*C. thermophilum*	24±1	374759	436	504
6:0 ENTH	*H. sapiens*	6±0	115971	20	249
5:0 ENTH *	*H. sapiens*	6±0	97358	12	153
6:6 ENTH/ANTH	*H. sapiens*	18±7	334702	290	5495
6:5 ENTH/ANTH *	*H. sapiens*	17±4	298960	81	3332

*determined in MS/MS experiments

Supplementary Table 3: SAXS Data collection and derived parameters for human ENTH.
Abbreviations: M_r: molecular mass; R_g: radius of gyration; D_{max}: maximal particle dimension; V_p: Porod volume; V_{ex}: Particle excluded volume.

	ENTH	ENTH + PIP2
**Data collection parameters**		
Instrument	EMBL P12 beam line (PETRA-III, DESY, Hamburg)	
Beam geometry	0.2 x 0.12 mm^2	
Wavelength (Å)	1.24	
s range (Å^{-1})[a]	0.01-0.46	
Exposure time (s)	1 (20×0.05 s)	
Concentration range (mg/mL)	0.4-1.5	0.4-1.5
Temperature (K)	288	288
**Structural parameters**[b]		
I(0) (cm^{-1}) [from *p(r)*]	0.012 ± 0.001	0.078 ± 0.001
R_g (Å) [from *p(r)*]	19 ± 1	34 ± 1
I(0) (cm^{-1}) (from Guinier)	0.012 ± 0.001	0.078 ± 0.001
R_g (Å) (from Guinier)	19 ± 1	35 ± 1
D_{max} (Å)	60	112
Porod volume estimate (Å^3)	34020 ± 10000	189000 ± 20000
Excluded volume estimate (Å^3)	17900 ± 2000	202000 ± 20000
Dry volume calculated from sequence (Å^3)[c]	22315/133782 (mon/hex)	
**Molecular-mass determination**		
I(0) (cm^{-1}) Glucose Isomerase (173,000 Da)	0.118 ± 0.001	
Molecular mass M_r (Da) [from *I(0)*]	17593 ± 2000	114356 ± 10000
Molecular mass M_r (Da) [from Porod volume ($V_p/1.6$)]	21263 ± 5000	118256 ± 10000
Molecular mass M_r (Da) [from excluded volume ($V_{ex}/2$)]	8950 ± 1000	101000 ± 10000
Calculated monomeric M_r from sequence (Da)	~18443	
**Software employed**		
Primary data reduction	RADAVER	
Data processing	PRIMUS/Qt	
Ab initio analysis	DAMMIF, DAMMIN	
Validation and averaging	DAMAVER	
Rigid-body modeling	CORAL	
Computation of model intensities	CRYSOL	
3D graphics representations	PyMOL, UCSF Chimera	

[a]Momentum transfer $s = 4\pi\sin(\theta)/\lambda$. [b]Values reported for 1.5 mg mL^{-1}. Dry volume determined using the server: http://www.basic.northwestern.edu/biotools/proteincalc.html

Supplementary Table 4 Amino acid sequences of ENTH and ANTH domains

Constructs		
Gene	Organism	Sequence
ENTH2	*S.Cerevisiae*	GAMGSMSKQFVRSAKNMMKGYSSTQVLVRDA<br>TANDSRTPSIDTLDDLAQRSYDSVDFFEIMDMLD<br>KRLNDKGKYWRHVAKSLTVLDYLVRFGSENCV<br>LWCRENFYVIKTLREFRHENESGFDEGQIIRVKA<br>KELVSLLNDEERLREERSMNTRNRRANRAAR
Sla2	*S.Cerevisiae*	GAMGSMSRIDSDLQKALKKACSVEETAPKRKH<br>VRACIVYTWDHQSSKAVFTTLKTLPLANDEVQL<br>FKMLIVLHKIIQEGHPSALAEAIRDRDWIRSLGR<br>VHSGGSSYSKLIREYVRYLVLKLDFHAHHRGFN<br>NGTFEYEEYVSLVSVSDPDEGYETILDLMSLQDS<br>LDEFSQIIFASIQSERRNTECKISALIPLIAESYGIY<br>KFITSMLRAMHRQLNDAEGDAALQPLKERYELQ<br>HARLFEFYADCSSVKYLTTLVTIPKLPVDAPDVF<br>LINDVDESKEIKFKKREPSVT
Sla2<br>(mut4A)	*S.Cerevisiae*	GAMGSMSRIDSDLQKALAKACSVEETAPARAA<br>VRACIVYTWDHQSSKAVFTTLKTLPLANDEVQL<br>FKMLIVLHKIIQEGHPSALAEAIRDRDWIRSLGR<br>VHSGGSSYSKLIREYVRYLVLKLDFHAHHRGFN<br>NGTFEYEEYVSLVSVSDPDEGYETILDLMSLQDS<br>LDEFSQIIFASIQSERRNTECKISALIPLIAESYGIY<br>KFITSMLRAMHRQLNDAEGDAALQPLKERYELQ<br>HARLFEFYADCSSVKYLTTLVTIPKLPVDAPDVF<br>LINDVDESKEIKFKKREPSVT
AP180	*H. Sapiens*	GAMSGQSLTDRITAAQHSVTGSAVSKTVCKATT<br>HEIMGPKKKHLDYLIQCTNEMNVNIPQLADSLFE<br>RTTNSSWVVVFKSLITTHHLMVYGNERFIQYLA<br>SRNTLFNLSNFLDKSGLQGYDMSTFIRRYSRYLN<br>EKAVSYRQVAFDFTKVKRGADGVMRTMNTEKL<br>LKTVPIIQNQMDALLDFNVNSNELTNGVINAAF<br>MLLFKDAIRLFAAYNEGIINLLEKYFDMKKNQC<br>KEGLDIYKKFLTRMTRISEFLKVAEQVGIDRGDI<br>PDLSQAPSSLLDALEQHLASLEGKK

HIP1R	*H. Sapiens*	GAMGSMNSIKNVPARVLSRRPGHSLEAEREQFD KTQAISISKAINTQEAPVKEKHARRIILGTHHEKG AFTFWSYAIGLPLPSSSILSWKFCHVLHKVLRDG HPNVLHDCQRYRSNIREIGDLWGHLHDRYGQLV NVYTKLLLTKISFHLKHPQFPAGLEVTDEVLEKA AGTDVNNIFQLTVEMFDYMDCELKLSESVFRQL NTAIAVSQMSSGQCRLAPLIQVIQDCSHLYHYTV KLLFKLHSCLPADTLQGHRDRFHEQFHSLRNFFR RASDMLYFKRLIQIPRLPEGPPNFLRASALAEHIK
ENTH1	*H. Sapiens*	GAMGSTSSLRRQMKNIVHNYSEAEIKVREATSN DPWGPSSSLMSEIADLTYNVVAFSEIMSMIWKRL NDHGKNWRHVYKAMTLMEYLIKTGSERVSQQC KENMYAVQTLKDFQYVDRDGKDQGVNVREKA KQLVALLRDEDRLREERAHALKTKEKLAQTA
Sla2	*C. Thermophilum*	GAMATTRSLDHAKAEAELAINIKKATSPEETAPK RKHVRSCIVYTWDHKSSLSFWAGLKVQPILADE VQTFKALITIHKVLQEGHPVTLREAMANRGWID SLSRGMMGEGVRGYGPLIREYVHFLLAKLSFHK QHPEFNGTFEYEEYISLKAIHDPNEGYETITDLMT LQDKIDQFQKLIFSHFRHIGNNECRISALVPLVAE SYGIYKFITSMLRAMHSSTGDNEALEPLRQRYD AQHYRLVKFYYECSNLRYLTSLITIPKL
ENTH	*C. Thermophilum*	GAMSKVIRSVKNVTKGYSSVQIKVREATSNDPW GPTGTQMSEIAQLTYGSSTDFYEIMDMLDKRLN DKGKNWRHVLKALKVMDYCLHEGSELVVTWA KKNIFIIKTLREFQYIDEEGRDVGQNIRVAARELT ALIQDEERLRAERNDRKMWKNRVNG
YAP1802	*S.Cerevisiae*	GAMGSMSSLYTKLVKGATKIKMAPPKQKYVDPI LSGTSSARGLQEITHALDIRLSDTAWTIVYKALIV LHLMIQQGEKDVTLRHYSHNLDVFQLRKISHTT KWSSNDMRALQRYDEYLKTRCEEYGRLGMDH LRDNYSSLKLGSKNQLSMDEELDHVESLEIQINA LIRNKYSVSDLENHLLLYAFQLLVQDLLGLYNA LNEGVITLLESFFELSIEHAKRTLDLYKDFVDMT EYVVRYLKIGKAVGLKIPVIKHITTKLINSLEEHL REETKRQRG

YAP1801	*S.Cerevisiae*	GAMGSMTTYFKLVKGATKIKSAPPKQKYLDPIL<br>LGTSNEEDFYEIVKGLDSRINDTAWTIVYKSLLV<br>VHLMIREGSKDVALRYYSRNLEFFDIENIRGSNG<br>SASGDMRALDRYDNYLKVRCREFGKIKKDYVR<br>DGYRTLKLNSGNYGSSRNKQHSINIALDHVESLE<br>VQIQALIKNKYTQYDLSNELIIFGFKLLIQDLLAL<br>YNALNEGIITLLESFFELSHHNAERTLDLYKTFV<br>DLTEHVVRYLKSGKTAGLKIPVIKHITTKLVRSL<br>EEHLIEDDKTHNTLITRAPPPPPLRSGC

Supplementary Table 5 Primers used to amplify ANTH and ENTH domain constructs

PCR Primers			
Gene	Organism	Forward primer	Reverse primer
Sla2	*C. thermophilum*	CAGGGCGCCATGGC<br>TACGACGCGCAGCC<br>TTGAT	GACCCGACGCGGTTA<br>AAGCTTCGGGATGGT<br>GATGAGGCTTGT
ENTH1	*C. thermophilum*	CAGGGCGCCATGTC<br>AAAAGTCATTCGAA<br>GT	GACCCGACGCGGTTA<br>GCCATTGACACGGTTC<br>TTCC
AP180	*H. sapiens*	CAGGGCGCCATGTC<br>TGGCCAGAGCCTGA<br>CG	GACCCGACGCGGTTA<br>TTTCTTTCCTTCCAAG<br>GAAGCTAAATG
Hip1R	*H. sapiens*	CAGGGCGCCATGA<br>ACAGCATCAAGAA<br>CGTG	GACCCGACGCGGTTA<br>TTCTTCCGGAATCACA<br>ACCACCGGCTTGATGT<br>GCTCAGCCAG

Supplementary References

1. Ford, M. G. *et al.* Simultaneous binding of PtdIns(4,5)P2 and clathrin by AP180 in the nucleation of clathrin lattices on membranes. *Science* **291,** 1051–1055 (2001).

2. Kabsch, W. & Sander, C. Dictionary of protein secondary structure: Pattern recognition of hydrogen-bonded and geometrical features. *Biopolymers* **22,** 2577–2637 (1983).
